# Editorial: Alzheimer-related affective symptoms – mechanism and treatment

**DOI:** 10.3389/fnagi.2023.1267304

**Published:** 2023-08-23

**Authors:** Keren Nitzan, Dan Frenkel, Matthew O. Parker, Ravid Doron

**Affiliations:** ^1^Department of Education and Psychology, The Open University, Ra'anana, Israel; ^2^Department of Neurobiology, The George S. Wise Faculty of Life Sciences, School of Neurobiology, Biochemistry and Biophysics, Tel Aviv University, Tel Aviv, Israel; ^3^Sagol School of Neuroscience, Tel Aviv University, Tel Aviv, Israel; ^4^Surrey Sleep Research Centre, School of Biosciences, University of Surrey, Guildford, United Kingdom

**Keywords:** depression, Levetiracetam, Alzheimer's disease, inflammation, cingulate cortex

## 1. Introduction

Alzheimer's disease (AD) is the most common cause of dementia and the fifth leading cause of death in adults older than 65. Aged individuals are disproportionately likely to suffer from anxiety, and this higher tendency is related to increased cognitive decline. In fact, aging is a prominent risk factor for AD and many of its comorbidities. AD is a progressive brain disorder that leads to neurodegeneration, cognitive deficits, and memory decline. In addition to the cognitive decline observed in Alzheimer's, there is a growing recognition of its comorbidities with other neurological, physical and psychological symptoms. These comorbidities include but are not limited to diabetes, cardiovascular disease, depression, and neuroinflammation (Santiago and Potashkin, 2021). In recent years, researchers have focused on investigating these comorbidities of AD to understand the disease progression better (Katabathula et al., 2023), identify potential new treatment approaches (Nitzan et al., 2021), and develop early biomarkers (Hu et al., 2023). In this Research Topic, we have delved into the different related symptoms of AD, aiming to enhance our understanding of disease etiologies and identify novel therapeutic approaches.

Aside from cognitive impairments, ~50% of AD patients experience some degree of depression or anxiety (El Haj et al., 2020). Neuropsychiatric symptoms such as depression, apathy and agitation are frequently observed in Alzheimer's patients (Banning et al., 2019). These symptoms can significantly impact the quality of life for both patients and their caregivers, as they may lead to increased distress and burden. To fully understand how emotional wellbeing and positivity affect age-related cognitive decline and dementia, it is crucial to thoroughly examine the connection between cognitive and affective health. In their article on this topic, Umucu et al. explore this mutually influential relationship. Their research supports previous studies that suggest a link between cognitive health and emotional wellbeing, particularly happiness. By studying a diverse group of individuals, they found that those with dementia reported less happiness than those with healthy cognitive functions. Interestingly, those with mild cognitive impairments (MCI) also reported lower happiness levels compared to cognitively healthy individuals and those with dementia. These findings shed important light on how cognitive decline impacts the emotional state of older adults and vice versa.

Social interaction plays a major part in emotional wellbeing and happiness. Indeed, research has shown that social interactions can positively impact dementia and depression (Hsiao et al., 2018). An article by Zhang et al. delves into this topic, exploring the correlation between social life (measured by the number of friends), depression, and cognitive decline. The study found an inverse relationship between the number of close friends and depression in elderly individuals with cognitive decline. This means that having more close friends can lower the risk of depression. However, a positive relationship was observed between depression and cognitive function change. In other words, the more severe the depression, the worse the decline in cognitive function. Interestingly, the study also found that the number of close friends affects the function of the right fusiform gyrus in individuals with cognitive decline. The authors suggest that changes in the functionality of this brain region can worsen depression, which in turn affects the function of the right occipital gyrus, ultimately leading to cognitive decline. This could be due to the fusiform gyrus's important role in face recognition, which is crucial for maintaining social relationships. Their findings could provide theoretical support for neuroimaging and help prevent and treat AD.

The alternation of neuronal networks is an integral part of AD pathology. Numerous studies identify irregularity in the function of various brain regions, such as the hippocampus, prefrontal cortex, and cingulate cortex (Palop and Mucke, 2016; Targa Dias Anastacio et al., 2022), which is intrinsically connected to understanding social cues (Millan et al., 2012). Using neuroimaging to study those abnormalities could uncover different pathological changes related to the various stages of AD progression. This is highly important as it may pave the path for a much-needed biomarker for early AD detection. With this goal in mind, Yuan et al. set out to predict the early stages of AD progression using neuroimaging to investigate functional connectivity changes in the anterior cingulate cortex (ACC). ACC is considered a component of the dorsal attention network, which is believed to be the foundation of neural aging. It is also involved in information processing and regulation and depression (Peng et al., 2021). Yuan et al. found that functional connectivity changes in the ACC subregions were distributed across different brain regions and could predict AD progression. Increased medial frontal gyrus (MFG) connectivity was positively correlated with executive function, while decreased posterior cingulate cortex (PCC) connectivity was positively correlated with memory. These findings provide new insights for clinical interventions across the preclinical AD spectrum.

The altered neuronal functions and hyperexcitability in various brain regions in AD could be related to epilepsy (Hsiao et al., 2018; Targa Dias Anastacio et al., 2022). Indeed, Zheng et al. show in their study that the anti-epileptic drug Levetiracetam can alleviate cognitive decline in an Alzheimer's disease mouse model. Furthermore, Levetiracetam enhanced the degradation and clearance of Aβ through activating autophagy and enhancing Aβ transport across the blood-brain barrier in treated AD-model mice. As mentioned above, inflammation plays a significant role in AD deficits. Interestingly, Levetiracetam suppressed neuroinflammation and neuronal loss by deactivating inflammasomes. Based on these findings, the authors suggest that the hyperexcitability seen in AD patients and animal models may be responsible for disrupting the processes required to encode new information during the acquisition or early phase of memory consolidation. The immune system is another critical factor related to AD and Neuropsychiatric symptoms. The role of immune system dysregulation and neuroinflammation in the development and progression of AD has been extensively studied (Palop and Mucke, 2016). Indeed, it was recently shown that immune-mediated disorders were significantly associated with an increased risk of dementia (Zhang et al., 2022). In their review, Huang et al. delve into the crucial issue of inflammatory factors in central system lesions, which can potentially cause neuronal damage or death by triggering a severe inflammatory cascade. They highlight the significance of neuroinflammation and pyroptosis as potential targets for AD therapy, as pyroptosis—a type of cell death mediated by inflammasomes and gasdermins—has been observed in AD patients and animal models. Studies have shown that inhibiting inflammasome-dependent pyroptosis can improve AD-related symptoms *in vitro* and *in vivo*. However, the connection between inflammation and AD pathogenesis may be more complex. Li et al.'s systematic review and meta-analysis evaluated the association between gout, the most common inflammatory arthritis in adults, and the risk of all-cause dementia. Interestingly, they suggest that gout may be a protective factor for AD, especially in patients with medication management. The authors propose that uric acid's pro- and antioxidant effects might be the underlying mechanisms for this effect, but further exploration is necessary.

In addition to comorbidities like depression, neuroinflammation, altered neuronal functions, cerebral ischemia and vascular diseases emerge as further noteworthy factors closely linked to AD (Zhang et al., 2021). The development of AD is intricately associated with vascular risk factors, and the onset of dementia can be hastened by cerebral ischemia. Mechanistically, oxidative and mitochondrial damage, inflammation, and hypoperfusion are common links between AD's pathophysiological features and ischemia. Therefore, these factors have been identified as potential targets for modifying and treating the disease. One promising therapeutic approach proposed by Elman-Shina and Efrati is Hyperbaric Oxygen Treatment (HBOT). Their thorough review explores the evidence of improved neurological function and quality of life in individuals with comorbidities like stroke, anoxic brain damage, traumatic brain injury, and vascular diseases following HBOT treatment. The cognitive benefits of HBOT are believed to stem from neuroplasticity mechanisms, including increased cerebral vascular flow and brain angiogenesis, axonal white matter regeneration and growth, stem cell proliferation, improved blood-brain barrier (BBB) integrity, and reduced inflammation. Thus, as Elman-Shina and Efrati suggest, Hyperbaric Oxygen Treatment (HBOT) presents a promising therapeutic approach to AD. Further research could improve outcomes and a better quality of life for individuals living with AD and its comorbidities by addressing cerebral ischemia and its association with vascular diseases.

To conclude, this Research Topic examines the intricate complexities surrounding AD and its comorbidities, providing valuable insights into different facets of the disease, enhancing our understanding of its progression and offering potential treatment avenues. Umucu et al. underscored the significance of happiness as a contributing factor in AD progression. Similarly, Zhang et al. emphasized the role of social life in AD, uncovering the neural mechanisms behind how social relationships affect. The exploration of early diagnosis methods was addressed by Yuan et al., who focused on utilizing neuroimaging techniques. Their study demonstrated the potential for identifying biomarkers that enable early detection by examining functional connection changes in various brain regions. In terms of therapeutic approaches, Zheng et al. delved into the benefits of addressing altered neuronal functions and hyperexcitability in the brain for AD treatment, focusing on epilepsy drugs. Their research provides insight into potential interventions that target these abnormalities, offering promising avenues for managing the disease. Furthermore, Huang et al. and Li et al. have also discussed the role of inflammation in AD. Lastly, Elman-Shina and Efrati's research delved into the potential etiologies of AD, with a particular focus on cerebral ischemia. Their comprehensive review examined interventions aimed at reversing brain ischemia.

Overall, the collective insights from these studies underscore the importance of comprehending the underlying mechanisms and treatments for Alzheimer's disease and its comorbidities. Understanding the underlying mechanisms and potential treatment options is pivotal in advancing our knowledge and developing effective interventions. Continued investment in research and collaborative efforts are crucial in our ongoing quest to unravel the complexities of AD and ultimately discover a cure for this devastating disease and help pave the way for a more personalized treatment approach based on these diseases that have significant comorbidity with AD. We hope this Research Topic will encourage further exploration and collaboration to understand and treat Alzheimer's disease.

## Author contributions

KN: Conceptualization, Writing—original draft, Writing—review and editing. DF: Writing—review and editing. MP: Writing—review and editing. RD: Supervision, Writing—review and editing.
